# Expression Atlas: gene and protein expression across multiple studies and organisms

**DOI:** 10.1093/nar/gkx1158

**Published:** 2017-11-20

**Authors:** Irene Papatheodorou, Nuno A Fonseca, Maria Keays, Y Amy Tang, Elisabet Barrera, Wojciech Bazant, Melissa Burke, Anja Füllgrabe, Alfonso Muñoz-Pomer Fuentes, Nancy George, Laura Huerta, Satu Koskinen, Suhaib Mohammed, Matthew Geniza, Justin Preece, Pankaj Jaiswal, Andrew F Jarnuczak, Wolfgang Huber, Oliver Stegle, Juan Antonio Vizcaino, Alvis Brazma, Robert Petryszak

**Affiliations:** European Molecular Biology Laboratory, European Bioinformatics Institute, EMBL-EBI, Hinxton, UK; Oregon State University, Corvallis, USA

## Abstract

Expression Atlas (http://www.ebi.ac.uk/gxa) is an added value database that provides information about gene and protein expression in different species and contexts, such as tissue, developmental stage, disease or cell type. The available public and controlled access data sets from different sources are curated and re-analysed using standardized, open source pipelines and made available for queries, download and visualization. As of August 2017, Expression Atlas holds data from 3,126 studies across 33 different species, including 731 from plants. Data from large-scale RNA sequencing studies including Blueprint, PCAWG, ENCODE, GTEx and HipSci can be visualized next to each other. In Expression Atlas, users can query genes or gene-sets of interest and explore their expression across or within species, tissues, developmental stages in a constitutive or differential context, representing the effects of diseases, conditions or experimental interventions. All processed data matrices are available for direct download in tab-delimited format or as R-data. In addition to the web interface, data sets can now be searched and downloaded through the Expression Atlas R package. Novel features and visualizations include the on-the-fly analysis of gene set overlaps and the option to view gene co-expression in experiments investigating constitutive gene expression across tissues or other conditions.

## INTRODUCTION

Since the last update in 2016, Expression Atlas (1) (http://www.ebi.ac.uk/gxa) at the European Bioinformatics Institute (EMBL-EBI) has been further enriched in data content and functionality. Its primary remit continues to be serving as a value-added database, providing information on where and under what conditions different genes are expressed on RNA and protein level. To achieve this, Expression Atlas enables gene- and condition-based queries across tissues, cell types, developmental stages, disease states and other conditions across multiple data sets. Expression Atlas aims to serve a wide research community by providing data sets from different organisms, including plants and metazoans.

Expression Atlas uses microarray and RNA-Seq data sets from ArrayExpress (2), NCBI’s Gene Expression Omnibus (GEO) (3), European Nucleotide Archive (ENA) (4) as well as other sources. Increasingly, mass spectrometry proteomics data coming from the PRoteomics IDEntification (PRIDE) database (5) are included. Criteria for selection of a gene expression data set for inclusion in Expression Atlas are: (i) the study must be of general interest; (ii) it must include at least three biological replicates and (iii) clear experimental variables must be available to enable re-analysis. All data sets are annotated by curators and the experimental variables are labelled with terms from a systematized ontology: the Experimental Factor Ontology (EFO) (6), which is also used by other EMBL-EBI resources. They are re-analysed in a standardized way, before they are loaded into the Atlas.

The data sets in Expression Atlas are classified either as *baseline* or *differential.* The baseline data sets report transcript or protein abundance typically in constitutive conditions, such as healthy tissues, cell types, developmental stages or cell lines. Baseline data sets are sourced from selected, high-quality RNA-Seq data sets, and in addition, several proteomics data sets have also been included recently. Differential studies report changes in expression between two different conditions, such as healthy and diseased tissue.

Since our last update, the number of studies in Expression Atlas has grown two-fold. A quarter of studies relate to plants, now spanning across twenty different species. The first multi-omics study including RNA-Seq and a proteomics data set in human induced pluripotent stem (iPS) cells, generated within the HipSci project (7), has been included recently. (https://www.ebi.ac.uk/gxa/experiments?experimentSet=HipSci). Expression Atlas has also analysed and will disseminate expression data from the Pancancer Analysis of Whole Genomes (PCAWG) project (https://www.ebi.ac.uk/gxa/experiments?experimentSet=Pan-cancer).

Since the last release, programmatic access has been made available through a new R package in Bioconductor (https://www.bioconductor.org/packages/release/bioc/html/ExpressionAtlas.html). Finally, users are now able to perform gene set overlap queries that result in a list of comparisons from differential experiments enriched for the group of genes of interest.

## RESULTS

### Data and analysis

#### Data

At the time of writing, Expression Atlas contains 3127 studies comprising 113 148 assays, including 1101 studies on human, 2193 on mammals (including human) and 731 studies in plants. Table 1 summarizes the top 15 organisms represented by the number of studies available in Expression Atlas. The data sets cover over 100 cell types from the Cell Ontology and over 700 diseases represented in the EFO. Although the majority of the data sets have been generated by the microarray technology (2587 studies), there are 537 studies based on RNA sequencing and 3 proteomics data sets. From the RNA-Seq data sets, 131 report baseline gene expression. Baseline data are now available for 32 species, with latest additions being pig (*Sus scrofa*), green monkey (*Chlorocebus sabaeus*), wild cabbage (*Brassica oleracea*), potato (*Solarum tuberosum*), zebrafish (*Danio rerio*), tomato (*Solanum lycopersicum*). The majority of studies continue to be of differential design, consisting of 2998 data sets, studying samples in 8391 differential comparisons, in 33 different organisms.

**Table 1.** Top 15 organisms in Expression Atlas—by the number of studies

Species	Number of differential studies	Number of baseline studies
*Homo sapiens*	1104	30
*Mus musculus*	875	36
*Arabidopsis thaliana*	513	6
*Rattus norvegicus*	135	2
*Drosophila melanogaster*	119	0
*Oryza sativa* Japonica Group	64	4
*Zea mays*	27	8
*Saccharomyces cerevisiae*	35	0
*Caenorhabditis elegans*	23	1
*Vitis vinifera*	16	4
*Gallus gallus*	18	2
*Glycine max*	13	6
*Solanum lycopersicum*	10	4
*Sus scrofa*	13	1
*Triticum aestivum*	8	4
Others	62	26

Since the previous update, Expression Atlas has processed and made available several additional large-scale baseline studies, such as Blueprint (8), which reports expression data across different types of sorted cells from human hematopoietic system (http://www.ebi.ac.uk/gxa/experiments?experimentSet=BLUEPRINT), HipSci (http://www.ebi.ac.uk/gxa/experiments?experimentSet=HipSci), Genotype-Tissue Expression (GTEx) version 6 (9), NIH Epigenomic Roadmap (10) (http://www.ebi.ac.uk/gxa/experiments/E-MTAB-3871), Human Developmental Biology Resource (HBDR) for prenatal brain development (11) (http://www.ebi.ac.uk/gxa/experiments/E-MTAB-4840), PCAWG—PanCancer Analysis of Whole Genomes Project (https://www.ebi.ac.uk/gxa/experiments?experimentSet=Pan-Cancer), which will be openly available with the PCAWG publication. Expression Atlas continues to re-annotate and re-analyse data sets with each new Ensembl (12) and Ensembl Genomes (13) release, therefore keeping all data sets up-to-date with the latest genome annotations.

#### Data dissemination

All data sets in Expression Atlas can be downloaded via FTP through a link on the website. Within each Experiment page, there are links to the downloadable files, such as tab-delimited RNA or protein quantification matrices, differential assay results, reports on protocols, as well as R data files. R files contain objects of ‘ExpressionSet’ class for microarray data sets and R objects of ‘RangedSummarizedExperiment’ class for RNA-Seq.

#### Expression Atlas R object

Since the previous update we have released an R package in Bioconductor, which enables users to search and download pre-packaged data from all studies in the Expression Atlas database into their R session (https://www.bioconductor.org/packages/release/bioc/html/ExpressionAtlas.html). Experiments can be searched via direct accessions, if known, or by using meta-data such as species, experimental conditions or tissues. The search uses ontology (based on EFO) term expansion, for instance, a search for ‘cancer’ will also retrieve experiments annotated as ‘leukemia’. Raw and normalized counts per experiment, as well as data annotations are available for download, for both RNA-Seq and microarray experiments.

#### Data analysis

Since the last update, there has been a change in the way RNA-Seq data sets are retrieved for processing in Expression Atlas. All RNA-Seq data sets from ENA, including the data from the Sequence Read Archive, SRA (14), corresponding to species covered by Expression Atlas are processed automatically using the open source iRAP pipeline and made available via the RNAseq-er Application Programming Interface (API) (15). This first level processing includes FASTQ QC, alignment, mapping QC and gene expression quantification. Selected experiments are then curated for dissemination in Expression Atlas and processed further downstream using quantile normalization followed by co-expression analyses (if baseline) or differential expression analysis, gene ontology and pathway enrichment (if differential). In addition, the Expression Atlas team actively looks for other RNA-Seq data sets that are not included in ENA for various reasons, such as controlled access, and try to obtain permissions to re-analyze and disseminate the processed data from these, and include them in the Atlas.

The tools employed for genome alignment, quantification and differential expression (Tophat2 (16)/Star (17), HTSeq (18), DESeq2 (19)) remain the same, as described in the previous update (1), although they have been updated to their latest versions. Additionally, users can now explore and download gene expression quantification values in TPM (transcripts per million) units as well as in FPKM (fragments per kilobase of exon model per million reads mapped).

All other basic methods for RNA-Seq and microarray analysis have remained the same as described previously (1). The analysis pipeline for microarray data has not changed since the last release. Proteomics data are currently processed in custom-designed way for each data set, using open source and/or free to use software such as OpenMS (20) and MaxQuant (21).

### New features on the user interface

#### Front end

The front page of Expression Atlas’ website has been updated. Different panels show the top six organisms in terms of numbers of studies available, including links to pre-constructed queries that allow a quick identification of these experiments. Similarly, landmark studies, such as GTEx, PCAWG and Blueprint are displayed as featured experiments, including links to their individual pages. Such enhancement allows a quick representation of some of the data content within Expression Atlas and enables users to quickly identify large projects or data sets from human and commonly used model organisms.

#### Gene set overlap queries

Expression Atlas now enables users to check if a set of genes of their interest overlaps significantly with the sets of differentially expressed genes in Atlas experiments of the same species. This enables users to test the results of their own experiments and investigate whether there is evidence that their set of differentially expressed (DE) genes is also DE in other experimental settings, involving similar or different experimental conditions, diseases or stresses. In a nutshell, the gene set overlap is implemented using the Fisher-exact test to check if the user's set of genes overlaps significantly with the DE genes (adjusted *P*-value ≤ 0.05) found in each comparison/study in the same species. The *P*-values from these tests are corrected for multiple tests using the Benjamini–Hochberg procedure. This functionality is available via the web-interface (front page) but also via a REST API. The implementation is available through https://github.com/gxa/atlas_gsa.

#### Genes with similar expression pattern

**Figure 1.**
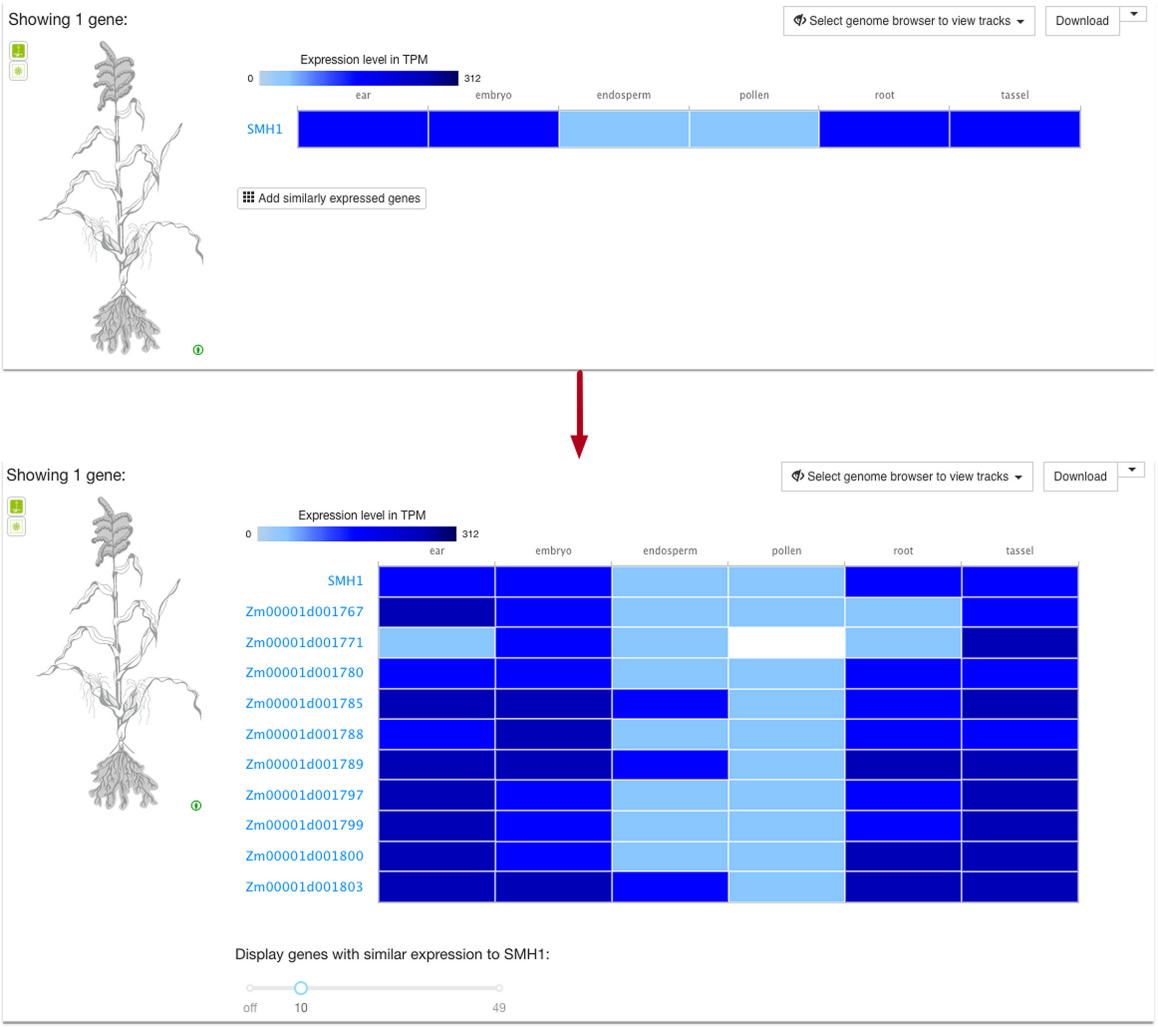
Exploring genes with similar expression pattern to SMH1 in six different tissues of *Zea mays*. (To reproduce the Figure, please click on ‘Add similarly expressed genes’ in https://www.ebi.ac.uk/gxa/experiments/E-MTAB-3826/Results?specific=true&geneQuery=%255B%257B%2522value%2522%253A%2522SMH1%2522%252C%2522category%2522%253A%2522symbol%2522%257D%255D&filterFactors=%257B%257D&cutoff=%257B%2522value%2522%253A0.5%257D&unit=%2522TPM%2522).

For each baseline experiment we now identify genes with similar expression patterns via the ClusterSeq R package (https://bioconductor.org/packages/release/bioc/html/clusterSeq.html). ClusterSeq (manuscript in preparation) investigates patterns of expression by comparing k-means clusters of gene expression across conditions, between each pair of genes, for different values of k. The results can be viewed through single gene queries within a baseline experiment, showing those similarly expressed genes to the original query, ranked by similarity of expression patterns (example in Figure 1).

#### New heatmap features

**Figure 2.**
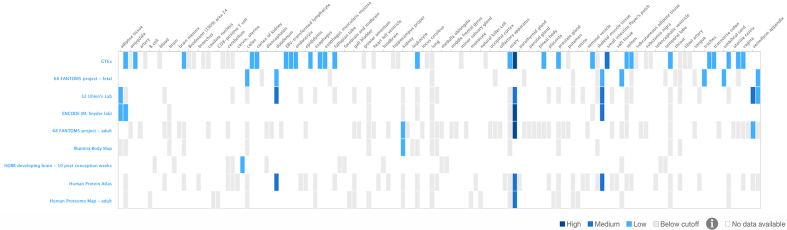
The new heatmap, includes more functionality for sorting and filtering data sets. Here, we show an example of the constituent expression of human gene REG1B across tissues in different baseline experiments (https://www.ebi.ac.uk/gxa/genes/ENSG00000172023).

Expression Atlas continues to visualize gene expression levels across different experimental conditions using a heatmap. Since the last update, the heatmap software library has been updated to enable larger data sets to be displayed, allowing for zooming in and resizing with a more aesthetically pleasing result. Additional features have been implemented to enable sorting of data according to expression levels across the different conditions. Furthermore, new filters allow users to select experimental factors of interest, based on groupings such as organs or anatomical systems (Figure 2).

#### Faceted search results

As the number of experimental studies included in Expression Atlas has significantly increased, we implemented new tools for filtering through the search results easily. Faceted search results in the baseline as well as the differential components enable the user to filter results by species, experimental condition, number of replicates and thus identify the most relevant results.

#### Baseline expression widget

Baseline expression data from Expression Atlas continue to be automatically included in Ensembl, Ensembl Genomes, Gramene (22), Ensembl Plants (23), Reactome (24) and Plant Reactome (25), via Javascript-based widgets. Since the last update, the baseline expression widget is also available through WormBase (26), the Complex Portal (27) and the Target Validation Platform by Open Targets (28). The widgets are easily accessible (https://github.com/gxa/atlas-heatmap) and can be integrated in any third-party site, provided that the bioentity identifiers match those of Expression Atlas.

## FUTURE DIRECTIONS

### Short term

#### Single cell RNA-Seq data sets

We are actively working on curation efforts, coupled with the development of analysis pipelines and appropriate visualization tools for single cell RNA-Seq data. In the future, we will release a new web-service to accommodate and visualize these data sets.

#### Dominant transcripts

We will be adjusting our analysis pipelines and web interfaces to process alternative transcript quantifications and visualize dominant transcripts for each gene (29).

#### Visualizing pathways

In the future, we will integrate pathway diagrams to enable users to view gene expression across the components of a pathway and how this is affected across different experimental conditions.

### Long term

#### Visualizing gene expression of orthologues and paralogues

Currently, through our keyword search, we enable users to implicitly view gene expression across orthologous genes, amongst other related genes. We aim to develop a query and visualization tool that will present orthologous gene expression explicitly in the user interface.

#### Meta-analyses

We will be testing methods for batch correction of expression data sets from different studies, with the view of releasing aggregated, cross-study results on baseline and differential views of the data.

#### Proteomics and metabolomics data sets

Future plans involve expanding the number of proteomics data sets included in Expression Atlas. We are developing analysis and QC pipelines and the additional needed infrastructure for enabling the routine integration of high-quality data sets, now that the availability of proteomics data in the public domain has become the norm (30,31). Furthermore, we will start to develop the necessary curation, data processing and visualization tools for including metabolomics data sets as well.

Finally, we are always seeking feedback from our users, through our website, training courses, conference attendance or usability testing. Plans for new features on our web-site and R package are continuously evaluated and adjusted through our interactions with our users.
